# Principles of Pathology and Practice of Medicine

**Published:** 1844-12

**Authors:** 


Art. VII. *Principles of Pathology and practice of Medicine.* By John Mackintosh, M.D., Lecturer on the practice of physic, Edinburgh, &c., &c., &c. Fourth American, from the last London edition. With notes and additions. By Samuel George Morton, M.D., formerly physician to the Philadelphia Hospital; author of Illuatrations of Pulmonary Consumption, &c., &c. Philadelphia, Lindsay & Blackiston. 1844. p.p. 892.

Whatever we may think of the practice of writing notes on the works of living authors, we cannot object to the annotations on the writings of the dead; among whom, unfortunately for practical medicine, the author of the work before us now reposes. But for such additions, books of sterling merit would soon fall "behind the times;" and go out of print, from not presenting, what so many readers are chiefly bent upon having—the latest discoveries and improvements. It would be well, that the latest *were* always the best; but, it is sometimes otherwise; for new modes of practice may supersede the old, without being superior or even equal to them. It would make a curious piece of history, to have all the different remedies, for any single disease, which have, from the beginning, been proposed, written down—each professing to be an improvement on the last, and received as such, although in many instances quite the reverse. Nevertheless, as medicine is a progressive science of observation and experience, all this seems unavoidable; for the spirit of innovation should not be quenched, and the real can only be distinguished from the unreal, by giving the whole a fair chance, in our trials.

We are far from intimating that the work before us, presents alterations rather than improvements; for, in truth, we regard it as one of the best systems extant. Its first merit is, that unlike some others, that of Dr. Good, or Dr. Craigie, for example, it is not overladen and smothered, with facts and opinions, drawn from a countless number of writers, ancient and modern: A second is the decisive tone in which the au thor expresses himself—decisive but not dogmatical: A third, we find, in the intelligible simplicity of his style: A fourth, in the sound practical judgment evinced in the treatment, which in most cases he recommends: A fifth, in the natural and excellent arrangement of his subjects. In his pathological speculations, our author shows himself familiarly acquainted with existing theories; and generally takes that which is most rational; keeping as clear of mere hypotheses as any other writer of the day.

The notes by Dr. Morton are sometimes critical; sometimes explanatory; more frequently they are interlarded additions to the text; designed to introduce the opinions or practice of American physicians, and put forth the results of his own ample experience. In discharging this duty, he seems to have been governed by a candid temper, in union with a matured judgment; and both student and physician will thank him for what he has added. He has enriched, without disfiguring, the text of his author.

In concluding this brief notice of a new edition of a standard work, we cannot withhold from the editor a different and higher tribute of respect. Although, but in middle life, and denied the advantages of perfect health, he has, by an industry of which in this country we have too few instances, found time, in the midst of his practice and his public teachings, to write a respectable work on Pulmonary Consumption, another on Anthropology, and so many papers on Geology, as to secure his election, not long since, to the presidency of the Association of American Geologists. Such diversified and successful labors, present him as an example which we hope may be extensively followed.

